# *Real-Time* insight into in vivo redox status utilizing hyperpolarized [1-^13^C] *N*-acetyl cysteine

**DOI:** 10.1038/s41598-021-90921-0

**Published:** 2021-06-09

**Authors:** Kazutoshi Yamamoto, Ana Opina, Deepak Sail, Burchelle Blackman, Keita Saito, Jeffrey R. Brender, Ronja M. Malinowski, Tomohiro Seki, Nobu Oshima, Daniel R. Crooks, Shun Kishimoto, Yu Saida, Yasunori Otowa, Peter L. Choyke, Jan H. Ardenkjær-Larsen, James B. Mitchell, W. Marston Linehan, Rolf E. Swenson, Murali C. Krishna

**Affiliations:** 1 Center for Cancer Research, National Cancer Institute, National Institutes of Health, Bethesda, MD 20892 USA; 2 Chemistry and Synthesis Center, National Heart, Lung, and Blood Institute, National Institutes of Health, Rockville, MD 20850 USA; 3 Department of Electrical Engineering, Technical University of Denmark, 2800 Lyngby, Denmark; 4 Radiation Biology Branch, Center for Cancer Research, National Cancer Institute, National Institutes of Health, Building 10, Room B3B35, Bethesda, MD 20892-1002 USA

**Keywords:** Analytical chemistry, Biophysical chemistry, Cancer imaging, Medical imaging, Imaging techniques, NMR spectroscopy, Biophysics

## Abstract

Drastic sensitivity enhancement of dynamic nuclear polarization is becoming an increasingly critical methodology to monitor *real-time* metabolic and physiological information in chemistry, biochemistry, and biomedicine. However, the limited number of available hyperpolarized ^13^C probes, which can effectively interrogate crucial metabolic activities, remains one of the major bottlenecks in this growing field. Here, we demonstrate [1-^13^C] *N*-acetyl cysteine (NAC) as a novel probe for hyperpolarized ^13^C MRI to monitor glutathione redox chemistry, which plays a central part of metabolic chemistry and strongly influences various therapies. NAC forms a disulfide bond in the presence of reduced glutathione, which generates a spectroscopically detectable product that is separated from the main peak by a 1.5 ppm shift. In vivo hyperpolarized MRI in mice revealed that NAC was broadly distributed throughout the body including the brain. Its biochemical transformation in two human pancreatic tumor cells in vitro and as xenografts differed depending on the individual cellular biochemical profile and microenvironment in vivo. Hyperpolarized NAC can be a promising non-invasive biomarker to monitor in vivo redox status and can be potentially translatable to clinical diagnosis.

## Introduction

Cells normally exist in a fine balance between reductive and oxidative states. When this balance is disrupted, either by external environmental stimuli or by abnormal metabolic states, the cellular integrity is compromised. To maintain the oxidative balance, the cells employ a variety of compartmentalized antioxidant systems to eliminate reactive oxygen species before damage can occur. Chief among these is glutathione/glutathione disulfide (GSH/GSSG) redox pair, which serves to maintain thiol redox balance through the NADPH-dependent reduction of glutathione disulfide (GSSG), and also serves as a primary control point in the coupled reactions that maintain intracellular redox balance^1–3^. In general, imbalance of redox state is also closely linked to the genesis and progression of numerous pathological conditions, including cancer, aging, diabetes, obesity, neurodegeneration, age-related retinopathy, cochlear degeneration, and chronic inflammatory diseases^1–4^. Particularly, malignant tumors frequently accumulate large amounts of glutathione as a countermeasure as the high rate of aerobic glycolysis found in many cancers can result in oxidative stress^5^.

There is therefore a strong interest in determining the GSH/GSSG balance in vivo. Furthermore, imaging redox environment of GSH/GSSH balance can be a powerful diagnostic strategy for non-invasively detecting cancer tissues, in particular, and assessing their early readout of therapeutic responses for ionizing radiation and some pharmaceuticals^6,7^. Measurements are complicated by the fact that glutathione is primarily intracellular and likely varies within a tumor due to metabolic heterogeneity^8,9^. As previously reported, ^13^C labeled dehydroascorbic acid has been used to probe the GSH/GSSG balance indirectly in preclinical studies^10–12^. Unfortunately, dehydroascorbic acid causes transient respiratory arrest at relatively low concentrations (10 mg kg^−1^) in mice^10^ as well as pancreatic toxicity^13^, conditions which may limit its translational potential. Hyperpolarized spin trap probes based on DMPO have been developed but are limited to detecting ROS production^14,15^. Toxicity concerns have also been expressed for the lanthanide based redox sensitive PARACEST MRI contrast agents^16,17^. Fluorescent techniques based either on the intrinsic fluorescence of NADH/NAD or specific probes for GSH/GSSH^18^ have proven effective for monitoring the redox environment preclinically and for tumors that lie close to the surface, for example melanoma and head and neck cancers^19^, but widespread adoption is hindered by the limited penetration of light in the visible/IR region of the EM spectrum^20^.

Here, we demonstrate *N*-acetyl cysteine (NAC)^21^, the acetylated derivative of the amino acid l-cysteine and a precursor of glutathione as a promising novel probe to monitor redox status which overcomes the potential safety disadvantages of dehydroascorbic acid^21–23^. We successfully designed stable ^13^C isotope labeled NAC with a long life time (*T*_*1*_ spin lattice relaxation) of hyperpolarization, and show tissue dependent redox transformation in human pancreatic tumor xenografts utilizing the cutting-edge technologies of both hyperpolarized [1-^13^C] NAC and metabolic ^13^C MRI, taking advantage of the drastic sensitivity enhancement ~ 10^5^ fold increase via hyperpolarization^24–26^. The biodistribution of hyperpolarized [1-^13^C] NAC and its biochemical transformation during the rapid imaging allows us to monitor important early reactions of thiol biochemistry in vivo.

## Results and discussion

**Figure 1:**
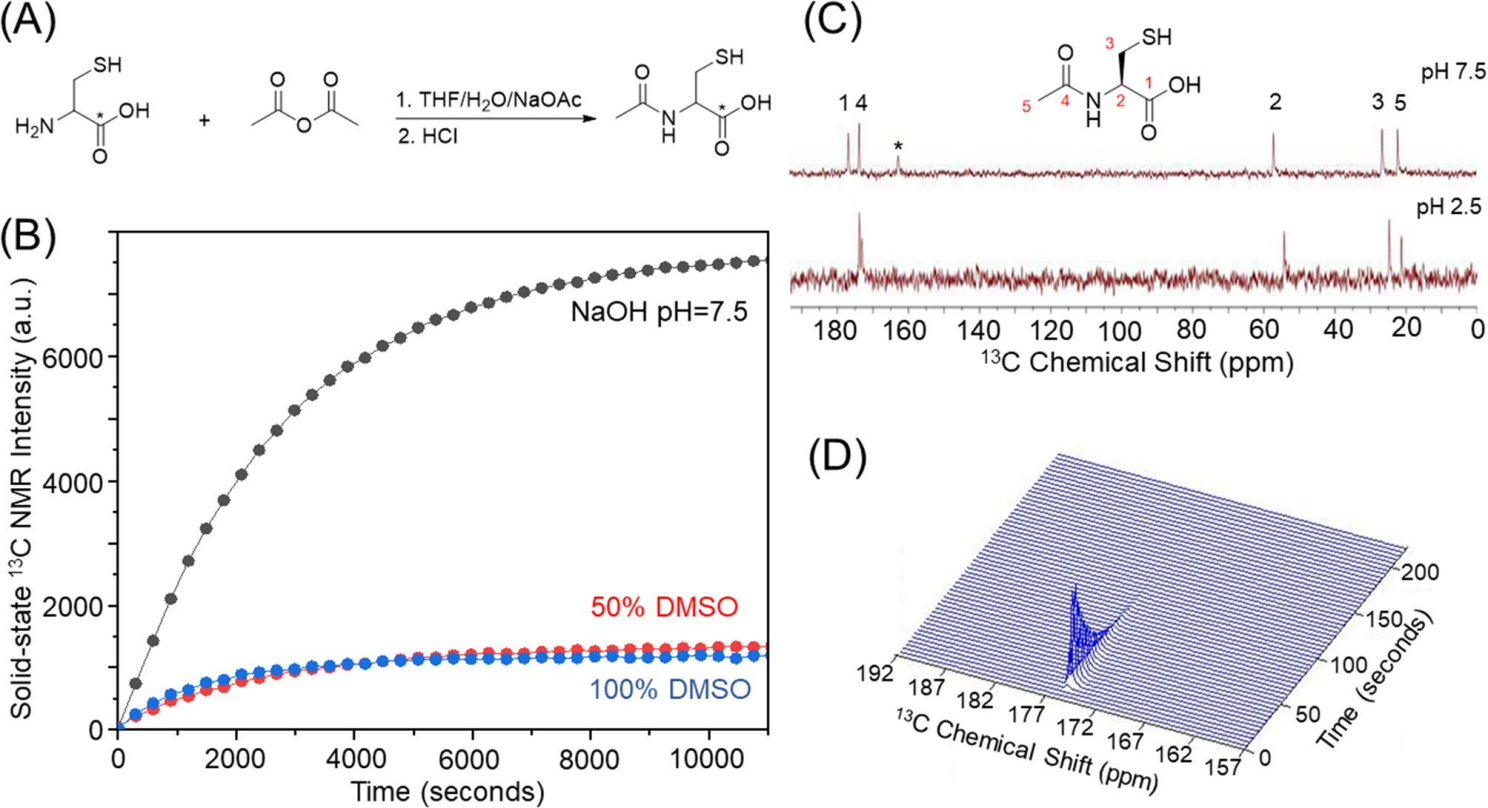
Optimizing sample conditions for hyperpolarized in vivo NMR/MRI experiments with *N*-acetyl cysteine. (**A**) Synthetic scheme of [1-^13^C] NAC. (**B**) Hyperpolarization build-up curves of [1-^13^C] NAC showing the drastic improvement of polarization using the optimized condition of a NaOH solution vs DMSO solutions. (**C**) ^13^C NMR spectra of unlabeled NAC at 1 T NMR confirm the pH dependence of polarization. An asterisk (*) is from a referencing standard of ^13^C Urea. (**D**) Dynamic spectra of hyperpolarized [1-^13^C] NAC in PBS buffer at 3 T MRI indicates a *T*_*1*_ relaxation time of 19.6 s.

Our preliminary hyperpolarized NMR experiments on natural abundance NAC indicated that only the [1-^13^C] NAC peak can be observed out of two potentially detectable carbonyl groups in NAC structure as shown in ^13^C NMR spectra (Supplementary Fig. S1), since the scalar relaxation from adjacent ^14^ N-nuclei shortens both the *T*_*1*_ and *T*_*2*_ relaxation times of the [4-^13^C] peak^27^. In addition to the relaxation characteristics, the [4-^13^C] position in NAC is more distal from the redox active sulfhydryl group, therefore, an efficient synthetic scheme was developed using commonly available starting materials to label NAC only in the [1-^13^C] position with relatively high yield by acetylation of [1-^13^C] L-cysteine (Fig. 1A). Briefly, [1-^13^C] l-cysteine was reacted with acetic anhydride in the presence of sodium acetate as the base in deoxygenated tetrahydrofuran^28^. Isolating the resulting product by crystallization was not successful as previously reported^29^, however the product could be purified by HPLC to afford [1-^13^C] NAC as a white, hygroscopic powder in 64% yield. The use of either HCl gas or concentrated aqueous HCl to convert the sodium salt to the free acid gave similar yields.

To validate [1-^13^C] NAC as an imaging probe, we first determined the sample conditions for polarization enhancement and the *T*_*1*_ longitudinal relaxation time in vitro. Samples using a standard solvent of DMSO^25^ polarized poorly (Fig. 1B), possibly because the anhydrous solvent favors the formation of intermolecular hydrogen bonds between two protonated carboxylic acid, which would increase the dipolar coupling associated with the carbonyl carbon and shorten *T*_*1*_ relaxation^30^. To reduce intermolecular association, [1-^13^C] NAC was titrated to a neutral pH of 7.5 using 5 M NaOH. The resulting solution of 3.2 M [1-^13^C] NAC became a homogenous self-glassing solution when frozen, a particular advantage for in vivo applications which typically require as highly concentrated solution as possible to achieve maximum sensitivity and to avoid complications from additives which may be potentially toxic or interfere with the metabolic processes being studied^31,32^. This polarizing solution shows efficient polarization build-up (Fig. 1C), reaching half of the equilibrium polarization in 11,000 s, similar to other hyperpolarized probes being considered for clinical use^25^. An improvement in polarization kinetics and equilibrium polarization values may be possible with optimization of polarization and glassing conditions^31,33,34^. This solution remained stable overtime at both neutral and acidic pH (Fig. 1(C)). The polarization was much weaker at pH 2.5, suggesting a possible role for hydrogen bonds among NAC clusters in reducing the equilibrium polarization^25,30^. The *T*_*1*_ relaxation time at 3 T of the 3.2 M [1-^13^C] NAC solution was determined to be 19.6 s by the decay dynamics of ^13^C MR signal (Fig. 1D).

**Figure 2:**
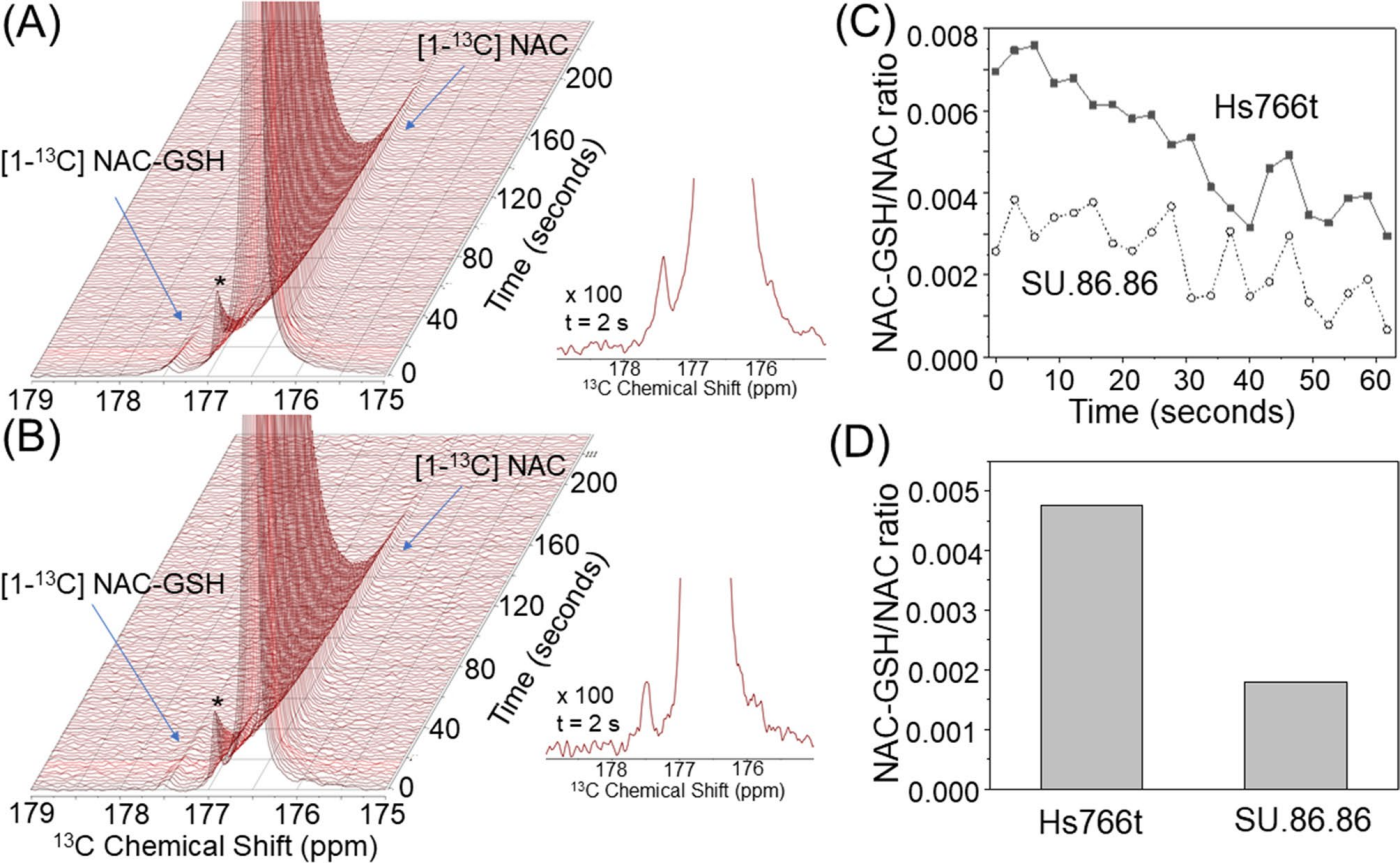
*Real-time* monitoring NAC metabolism in *in cell* NMR spectroscopy of tumor cell lines. *In cell* dynamic ^13^C NMR spectra of hyperpolarized [1-^13^C] NAC at 1 T NMR on 20 × 10^6^ cells of human pancreatic tumor cell lines of Hs766t (A, left) and SU.86.86 (B, left). Expanded spectra with 100 times magnifications at 2 s after the hyperpolarized [1-^13^C] NAC injections in Hs766t (**A**, right) and SU.86.86 (**B**, right) cells. (**C**) Time dependence of NAC-GSH/NAC peak intensity ratio after mixing HP-NAC with PDAC cells. (**D**) Comparison of the ratios of NAC-GSH to NAC between Hs766t and SU.86.86 cell lines. A chemical shift peak around 177 ppm indicated with asterisk (*) is assigned as the dimeric form of NAC.

**Figure 3:**
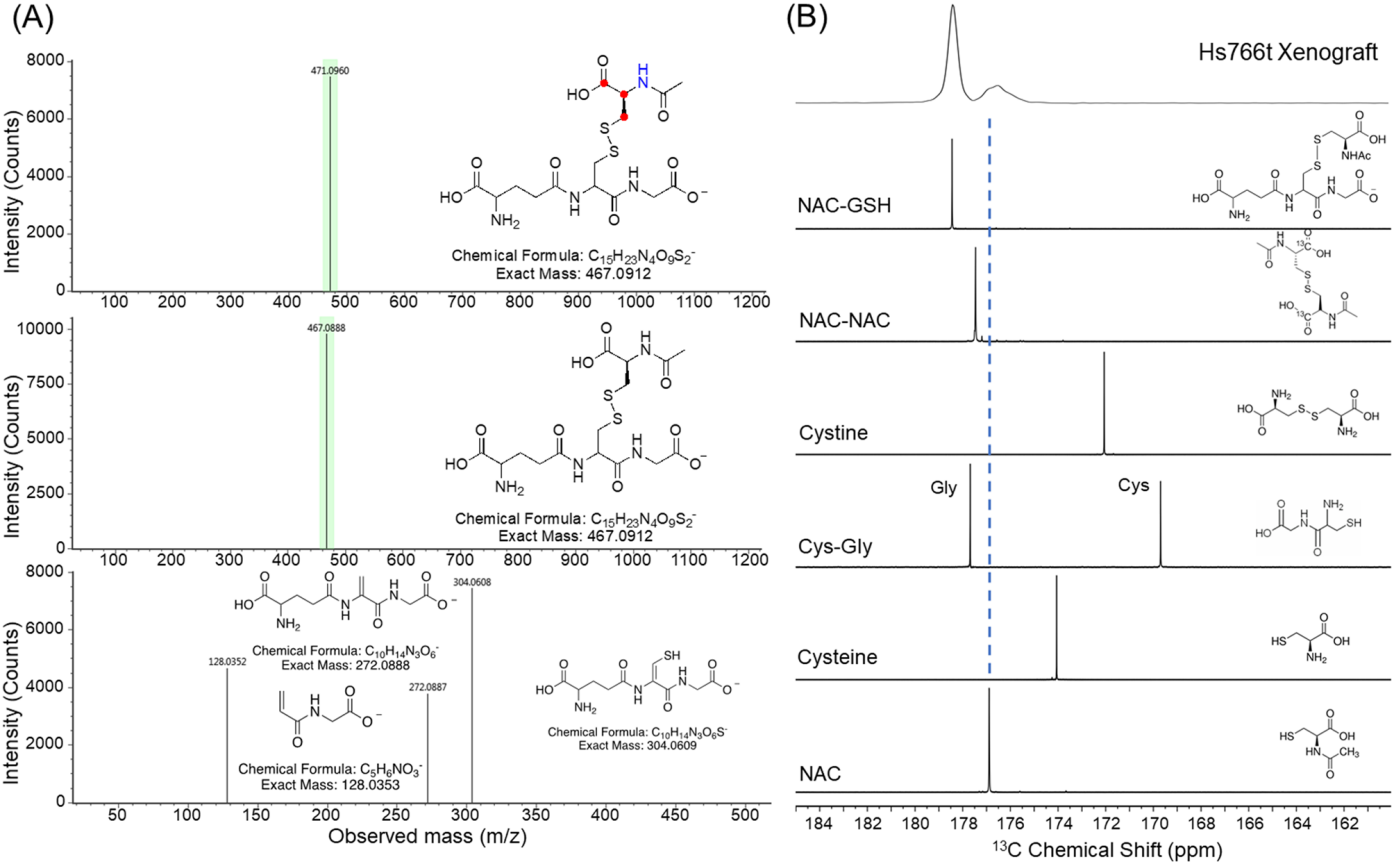
Identification of products from hyperpolarized [1-^13^C] NAC. (**A**) ESI–MS spectra of SU.86.86 tumor extracts with (top) and without (middle) isotope labeling in NAC. ^13^C labeled atoms are indicated in red, ^15^N labeled atoms are indicated in blue in the NAC-GSH structure. High energy ESI–MS spectrum of NAC-GSH with possible fragment identifications (bottom). (**B**) ^13^C NMR spectra of synthesized model compounds at 400 MHz, pH 7.5 that represent potential products in comparison to the spectrum from the hyperpolarized [1-^13^C] NAC MRS experiments in Hs766t tumor xenograft at 20 s after the *iv* injection (top).

These excellent optimized conditions allowed us to use [1-^13^C] NAC for *in cell* NMR and in vivo MRI. *In cell* dynamic ^13^C NMR spectra of hyperpolarized [1-^13^C] NAC at 1 T NMR spectrometer on human pancreatic ductal adenocarcinoma (PDAC) cell lines, which have one of the worst prognoses among common cancers and need effective diagnostic approaches^35–37^, Hs766t (Fig. 2A) and SU.86.86 (Fig. 2B), in both cases showed three distinct peaks, a major peak at 176.5 ppm and two peaks at 176.8 and 177.5 ppm. The major peak was immediately identified as [1-^13^C] NAC on the basis of the ^13^C NMR spectrum of a pure phantom sample (Fig. 3B). The peak at 176.8 ppm was assigned as an oxidized NAC-NAC dimer in a similar manner (Fig. 3B, Supplementary Figs. S4 and S5)^22^. The peak at 177.4 ppm was tentatively identified as the oxidized NAC-GSH dimer based on the ^13^C NMR spectrum of an authentic sample (Fig. 3(B), Supplementary Fig. S6). To confirm this assignment, metabolomics approaches based on Mass Spectrometry (MS) were used. Tumor xenografts were treated with unlabeled and [^13^C_3_,^15^ N]-labeled NAC, extracted according to published protocols and analyzed by LC/MS. The data were collected using scanning quadrupole data-independent acquisition, which gives fragmentation information for precursor peaks to aid in identification. The NAC metabolite was traced by first identifying retention times (rt) and m/z pairs which are unique to the labeled sample relative to the unlabeled sample and therefore indicate conversion products of the labeled probe (Fig. 3A). Peaks shifted by 4 Da with identical retention times correspond to labeled products. A 471/467 m/z pair with a rt of 4.47 min confirmed the third product at 177.4 ppm was the oxidized NAC-GSH dimer, which was further supported by fragmentation analysis (Fig. 3A).

Figure 2 indicates hyperpolarized [1-^13^C] NAC can produce NAC-glutathione (NAC-GSH) in cell cultures. The rapid kinetics of this reaction suggest that hyperpolarized NAC can permeabilize through cell membranes without active transport^38,39^, and chemical reactions of hyperpolarized NAC with GSH can be observed within the lifetime of this hyperpolarized ^13^C probe. The time-dependence of the NAC-GSH/NAC peak intensity ratio after mixing hyperpolarized NAC with human PDAC cell lines (Fig. 2C) and the area under the curve ratio (Fig. 2D) suggest a higher potential for NAC oxidation with glutathione in SU.86.86 cells. The potential for NAC to be oxidized by glutathione depends on the GSH/GSSG balance, as NAC is not oxidized by GSH^40^. Lower concentrations of NAC-GSH in SU.86.86 is consistent with previous metabolomics experiments^41,42^, as the reliance of SU.86.86 on the TCA cycle depletes NAD^+^ and therefore shifts the equilibrium of the GSH/GSSG redox buffer system towards GSH.

Furthermore, to test the effectiveness of [1-^13^C] NAC as an imaging probe in vivo, real-time dynamic ^13^C MR spectra of hyperpolarized [1-^13^C] NAC were acquired from mice bearing tumor xenograft. We first conducted ^13^C two-dimensional chemical shift imaging (CSI) experiments in both a healthy mouse body and head after intravenous *(iv*) injection of hyperpolarized [1-^13^C] NAC solution through a tail vein cannula as shown in Supplementary Fig. S2. Hyperpolarized [1-^13^C] NAC was globally distributed throughout the mouse body within 30 s after the injection of hyperpolarized solutions, with higher concentrations of [1-^13^C] NAC in the liver, kidney, and heart region. Conversely, lower signal was observed in the lung region (Supplementary Fig. S2A). Although the blood–brain barrier (BBB) permeability of NAC is subject to controversy, the presence of hyperpolarized [1-^13^C] NAC in the normal mouse brain indicates the possibility that membrane-permeable NAC may penetrate the blood–brain barrier and be retained in the brain (Supplementary Fig. S2B)^38^. However, the current experimental design cannot distinguish intercellular or interstitial conversion from partial volume effects arising from circulating NAC in blood vessels and this interpretation should be viewed with caution in light of the fast timescales involved^38,43^.

**Figure 4:**
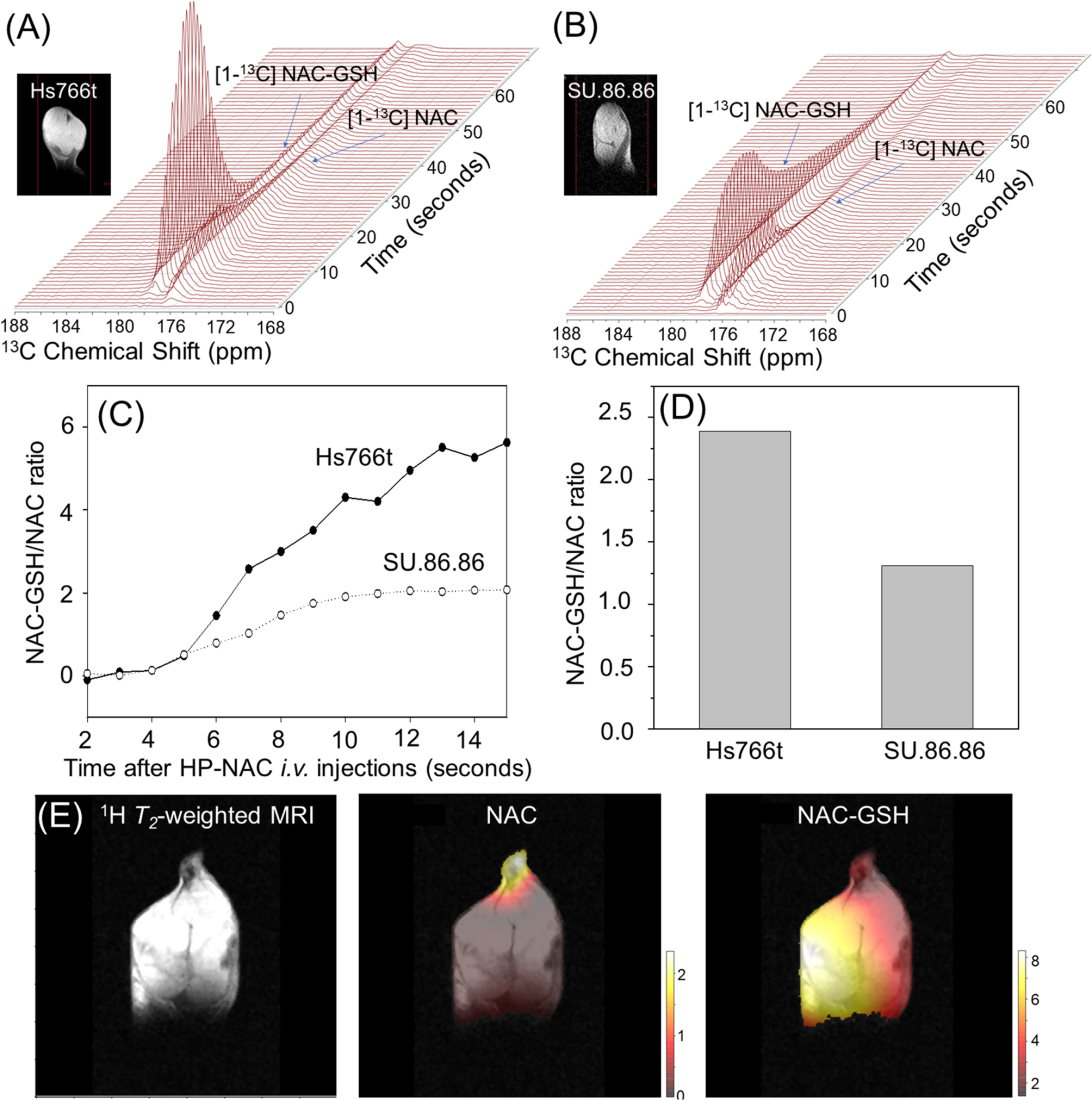
*Real-time* observation of NAC metabolism effectively probes redox status in tumors in vivo. Dynamic ^13^C MR spectra of hyperpolarized [1-^13^C] NAC at 3 T MRI on human pancreatic tumor xenografts of Hs766t (**A**) and SU.86.86 (**B**). Differences in the conversions reflect the redox status of each tumor. (**C**) Time dependence of NAC-GSH/NAC peak intensity ratio after the *iv* injection of HP-NAC. (**D**) Comparison of the ratios of NAC-GSH to NAC between Hs766t and SU.86.86 tumor xenografts. (**E**) Site-specific differences in chemical conversions of hyperpolarized [1-^13^C] NAC by ^13^C Chemical shift imaging in Hs766t xenografts.

Metabolites of in vivo hyperpolarized [1-^13^C] NAC were not observed in the liver and kidney regions of these normal mice, suggesting that the enzymatic conversion of NAC was below the detection level in the absence of any imposed oxidative stress either focally or globally, although in vitro enzymatic assays of hyperpolarized NAC incubated with acylase 1 resulted in immediate production of cysteine (Supplementary Fig. S3). To test [1-^13^C] NAC in a tumor environment, mouse leg xenografts of Hs766t and SU.86.86 were prepared. The single voxel MRS signal for NAC-GSH is much stronger in the xenografts (Fig. 4A,B), consistent with higher cellular density in vivo. In other aspects, the in vivo data (Fig. 4C,D) resembles the in vitro data of the corresponding cell cultures (Fig. 2C,D). Similar to the in vitro results, NAC-GSH is rapidly formed in both tumors and the amount of NAC-GSH formed is higher in Hs766t than in SU.86.86 tumors. Encouraged by these results, we also confirmed that NAC-GSH formation could be imaged as shown in Fig. 4E. Using chemical shift imaging, it can be seen that NAC-GSH formation is highest in the tumor and lowest in the surrounding muscle and leg regions while the distribution of non-converted NAC was observed dominantly in the leg area, which is consistent with higher overall glutathione concentrations in the tumor regions (Fig. 4)^41,42^.

Interestingly, the ^13^C chemical shifts of both NAC and its reaction products, NAC-GSH, have a pH dependence (Supplementary Fig. S7), although this may require a high field magnet and/or well optimized shimming conditions to adequately resolve. This could be advantageous to identify the site-specific differences in pH, in heterogeneous tumors or differentiate the components inside and outside of tumor cells on the experiments with high field magnet with optimized shimmed conditions. Using suitable animal disease models, and clinical/biological targets, hyperpolarized [1-^13^C] NAC can be used to probe the enzymatic activities or oxidative stress throughout the body. As for practical clinical subjects, NAC is a widely used in clinical practices as a beneficial antioxidant and suggested as a potential therapeutic agent in the treatment of cancer, drug toxicity, heart disease, HIV infection, cystic fibrosis, liver detoxification, Alzheimer disease, diabetes, and other diseases related to oxidative stress^22^.

## Conclusions

In summary, we have demonstrated the application of a novel hyperpolarized ^13^C probe based on a widely used, FDA-approved pharmaceutical agent to assess oxidative stress in human tumor cells and xenografts non-invasively utilizing hyperpolarized ^13^C MRS imaging. State-of-the-art hyperpolarized ^13^C MRS allows us to obtain real-time monitoring of in vivo physiological process and progression of diseases through changes in metabolic flux^7,8,13–16,28^. As we have described in this study, in order to design effective in vivo hyperpolarized MRI probes, successful hyperpolarized isotope labeled biomolecules have to exhibit the following requirements: (a) suitable biocompatibility and nontoxicity, (b) the availability of an organic synthesis scheme for the production of isotope labeled probes at high yields, (c) long spin lattice *T*_*1*_ relaxation times, (d) efficient nuclear spin polarization with high concentrations of substrates, (e) the ability to monitor biologically or clinically relevant mechanisms of metabolic pathways and/or physiological processes, (f) rapid distribution of the hyperpolarized probes to the targeted imaging regions, (g) adequate chemical shift differences between original injected substrates and metabolic products, (h) detectable MR signals in both injected probes and the products. We demonstrate here hyperpolarized NAC potentially satisfies all of these requirements. The biodistribution of hyperpolarized [1-^13^C] NAC demonstrates that significant signal levels can be observed in globally in a mouse, including the heart, liver, kidneys, brain, muscle, and lungs. Hyperpolarized [1-^13^C] NAC can potentially be used for probing free radical scavengers, antioxidant, and enzymatic activities, including acylases, which catalyze the deacetylation of NAC to produce cysteines (Supplementary Fig. S3). Although cysteine-containing NAC has been also considered as a precursor of glutathione, in this study the formation of GSH was not detectable in our hyperpolarized MR spectra, most likely due to the relatively short observation window in hyperpolarized experiments and the possible indirect mechanism of the GSH synthesis after *iv* injections in in vivo^44^. Further studies to investigate the detailed metabolic pathways of hyperpolarized ^13^C NAC using deuterated analogs to enhance *T*_*1*_ relaxation time and other metabolomics approaches are in progress in our laboratory. These findings in this study can promote strategic labeling schemes of biocompatible pharmaceuticals for hyperpolarized MRI to monitor key metabolic reactions. Our current work expands the hyperpolarization of FDA-approved pharmaceutical compounds to image in situ metabolic activities and/or MRI contrast agents, which may be relatively smoothly translatable to high impact clinical applications with proven biocompatibilities.

## Methods

### Synthesis of [1-^13^C] N-acetyl cysteine

All commercially available reagents were used as received unless otherwise noted. [1-^13^C] l-cysteine and D_2_O were purchased from Cambridge Isotope Laboratories, Inc (Tewksbury, MA). Liquid chromatography mass spectrometry (LC–MS) was performed on an Agilent 1200 Series Mass Spectrometer equipped with LC/MSD TrapXCl Agilent Technologies instrument. Preparative RP-HPLC analysis was performed on an Agilent 1200 Series instrument equipped with a multi-wavelength detector. ^1^H and ^13^C-NMR were recorded on a Varian 400 MHz NMR spectrometer.

### N-Acetyl cysteine-[1-^13^C] 1

[1-^13^C] l-cysteine **2** (0.50 g, 4.1 mmol) and sodium acetate 
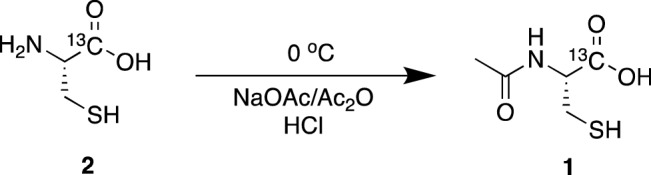
trihydrate (1.11 g, 8.2 mmol) was dissolved in a degassed THF: water (90:10 v/v, 10 mL) solution and was stirred at room temperature for 20 min under nitrogen. The reaction was cooled to 0 °C and acetic anhydride (0.44 g, 4.3 mmol) was added dropwise. The reaction was stirred for 16 h at room temperature under nitrogen. The clear solution was cooled and acidified to pH 1 with concentrated HCl. The solvent was evaporated *in vacuo* and the product purified by RP-HPLC. Purification was performed using an Agilent Prep C18 column (5 µm, 50 × 100 mm) with a flow rate of 50 mL/min. A linear gradient of 5–35% acetonitrile with 0.1% TFA was used to elute the product **1** as a white, hygroscopic powder after lyophilization (0.41 g, 64%). ^1^H-NMR (400 MHz, D_2_O): δ 2.08 (3H, s, CH_3_), 2.99 (2H, m, CH_2_SH), 4.63 (1H, m, NHCH). ^13^C-NMR (400 MHz, D_2_O): δ 23.45 (CH_3_), 27.41 (CH_2_SH), 57.51 (d, ^1^J_C-C_ = 232 Hz, NHCH), 173.66 (CH_3_C = O), 176.89 (COOH). *m/z* (ESI–MS +): 165.0 [M+H]^+^.

### Hyperpolarized ^13^C MRI

NaOH (5 M) was added to [1-^13^C] NAC powder and OX063 to produce a 3.2 M [1-^13^C] NAC solution with 17 mM OX063 at pH of 7.5. 35 mL of 3.2 M [1-^13^C] NAC with 17 mM OX063 was hyperpolarized using the SPINlab (GE Healthcare) for 3–4 h, and the scans were performed using the Philips Achieva 3 T MRI. ^13^C two dimensional spectroscopic chemical shift images (CSIs) were acquired with a 28 × 28 mm, field of view in a 10 mm axial slice through the head, a matrix size of 14 × 14, spectral width of 3333 Hz, repetition time of 86 ms, and excitation pulse width a flip angle of 3° for the mouse head, and with a 32 × 32 mm, field of view in a 10 mm coronal slice through the body, a matrix size of 16 × 16, spectral width of 3333 Hz, repetition time of 85 ms, and excitation pulse with a flip angle of 10° for the mouse body. CSIs were acquired 30 s after the beginning of the hyperpolarized [1-^13^C] NAC injections.

### LC/MS methods for identification of product

Chemicals: [^13^C_3_, ^15^N]-NAC was purchased from Cambridge Isotope Laboratories, Inc (Tewksbury, MA). NAC, formic acid and ammonium formate was purchased from Sigma-Aldrich (St. Louis, MO). LC–MS acetonitrile was purchased from Fisher Scientific. Liquid chromatography/mass spectrometry analysis was performed on a Waters Acquity UPLC coupled to a Waters Xevo Q-ToF quadruple time of flight mass spectrometer operating in electrospray ionization (ESI) in negative mode. The capillary and sampling cone voltages were set to 1.5 kV and 10 V, respectively. Source and desolvation temperatures were set to 120 °C and 450 °C, respectively, and the cone and desolvation gas flows were set to 50.0 and 800.0 L/h, respectively. To maintain mass accuracy, leucine enkephalin was used at a concentration of 2 ng/mL in 50:50 acetonitrile/water containing 0.1% formic acid and injected at a rate of 10 μL/min. Data was acquired using SONAR (scanning quadrupole data-independent acquisition) in continuum mode. In low-energy MS1 mode, the quadrupole was scanned between 50 -1200 m/z, with a quadrupole transmission width of ~ 50 Da, with a collision cell energy of 10 eV. In high-energy MS2 mode, the collision cell energy was ramped between 20 and 30 Da. The analytes were separated by HILIC chromatography on an Xbridge BEH Amide (2.5 μm, 2.1 × 100 mm) column. Chromatographic separation was achieved with 95:5 water:acetontrile containing 10 mM ammonium formate, pH 3 (A) and 95:5 acetonitrile:water containing 10 mM ammonium formate, pH 3 (B). Gradient elution, with a flow rate of 0.340 mL/min, began at 95% B, then decreased to 50% B from 0.0 to 3.4 min, 50–5% B from 3.4 to 5.39 min, held at 5% B from 5.39 to 6.37 min, then returned to initial conditions (95%B) in 0.20 min. The column was equilibrated at 95% B for 4.43 min before the next injection. The column temperature was maintained at 40 °C in a column oven.

### Cell culture and animal studies

All of the animal experiments were conducted in compliance with the Guide for the Care and Use of Laboratory Animal Resources and ARRIVE guidelines, and experimental protocols were approved by the Animal Care and Use Committee, National Cancer Institute (NCI-CCR-ACUC)^45,46^. The human pancreatic ductal adenocarcinoma (PDAC) cell lines, Hs776t, and SU.86.86 cells, were purchased from Threshold Pharmaceuticals (Redwood City, CA). Human pancreatic tumor inoculated mice were generated by subcutaneous injection of 3 × 10^5^ cells into the right hind legs of mice. Detailed conditions for cell culture and xenograft tumor development were as described previously^47^. Athymic nude mice were obtained from the Frederick Cancer Research Center, Animal Production (Frederick, MD). Both respiration (60–90 breaths per min) and temperature (35–37 °C) were maintained at a normal physiological range and monitored continuously during the animal experiment using the adjusted anesthesia, isoflurane.

### Extraction of metabolites from tumors

^13^C, ^15^N labeled NAC ([^13^C_3_, ^15^N] cysteine) was purchased from Cambridge Isotope Laboratories, Inc (Tewksbury, MA). Unlabeled NAC was purchased from Sigma-Aldrich (St. Louis, MO). 2.76 mg of either ^13^C, ^15^N labeled NAC ([^13^C_3_, ^15^ N] cysteine) or unlabeled NAC was intravenously injected to track metabolites of NAC in xenograft tumors. Mice were euthanized in 2 min after the tail vein injections. The tumors were rapidly removed and flash frozen in the liquid nitrogen, then they were stored at − 80 °C. The metabolites were extracted from the obtained tumors using a previously reported procedure^48^. The resulting lyophilized aqueous metabolite extracts were used for the MS for metabolomic analysis.

## Supplementary Information

Supplementary Information.
