# Supplementary material for: Real-Time insight into in vivo redox status utilizing hyperpolarized [1-13C] N-acetyl cysteine
Source: Sci Rep. 2021 Jun 9;11:12155. doi: 10.1038/s41598-021-90921-0 (PMC8190077; doi:10.1038/s41598-021-90921-0)
Supplement: Supplementary file 1 — Supplementary Information. [file 41598_2021_90921_MOESM1_ESM.docx]

**Supporting Information**

*Real-time* Insight into *In Vivo* Redox Status utilizing Hyperpolarized [1-^13^C] *N*-Acetyl Cysteine

Kazutoshi Yamamoto,^1^ Ana Opina,^2^ Deepak Sail,^2^ Burchelle Blackman,^2^ Keita Saito,^1^ Jeffrey R. Brender,^1^ Ronja M. Malinowski,^3^ Tomohiro Seki,^1^ Nobu Oshima,^1^ Daniel R. Crooks,^1^ Shun Kishimoto,^1^ Yu Saida,^1^  Yasunori Otowa,^1^  Peter L. Choyke,^1^  Jan H. Ardenkjær-Larsen,^3^  James B. Mitchell,^1^ W. Marston Linehan,^1^ Rolf E. Swenson,^2^ and Murali C. Krishna^1*^

^1^ Center for Cancer Research, National Cancer Institute, National Institutes of Health, Bethesda, MD, 20892 United States

^2^ Chemistry and Synthesis Center, National Heart, Lung, and Blood Institute, Rockville, MD, 20850 United States

^3^ Department of Electrical Engineering, Technical University of Denmark, Lyngby, 2800 Kgs. Denmark

* Corresponding author email address: cherukum@mail.nih.gov

Table of Contents:

**Materials and Methods** 2

**Supplemental Figures S1-S8** 5 - 13

**References** 14

**Materials and Methods**

*Synthesis of [1-^13^C] N-acetyl cysteine*

All commercially available reagents were used as received unless otherwise noted. [1-^13^C] L-cysteine and D_2_O were purchased from Cambridge Isotope Laboratories, Inc (Tewksbury, MA). Liquid chromatography mass spectrometry (LC-MS) was performed on an Agilent 1200 Series Mass Spectrometer equipped with LC/MSD TrapXCl Agilent Technologies instrument. Preparative RP-HPLC analysis was performed on an Agilent 1200 Series instrument equipped with a multi-wavelength detector. ^1^H and ^13^C-NMR were recorded on a Varian 400 MHz NMR spectrometer.

**N-Acetyl cysteine-[1-^13^C] 1:** [1-^13^C] L-cysteine **2** (0.50 g, 4.1 mmol) and sodium acetate trihydrate (1.11 g, 8.2 mmol) was dissolved in a degassed THF: water (90:10 v/v, 10 mL) solution and was stirred at room temperature for 20 min under nitrogen. The reaction was cooled to 0 ^o^C and acetic anhydride (0.44 g, 4.3 mmol) was added dropwise. The reaction was stirred for 16 h at room temperature under nitrogen. The clear solution was cooled and acidified to pH 1 with concentrated HCl. The solvent was evaporated *in vacuo* and the product purified by RP-HPLC. Purification was performed using an Agilent Prep C18 column (5 µm, 50 x 100 mm) with a flow rate of 50 mL/min. A linear gradient of 5-35 % acetonitrile with 0.1% TFA was used to elute the product **1** as a white, hygroscopic powder after lyophilization (0.41 g, 64%). ^1^H-NMR (400 MHz, D­­_2_O): δ 2.08 (3H, s, CH_3_), 2.99 (2H, m, CH_2_SH), 4.63 (1H, m, NHCH). ^13^C-NMR (400 MHz, D_2_O): δ 23.45 (CH_3_), 27.41 (CH_2_SH), 57.51 (d, ^1^J_C-C_ = 232 Hz, NH­CH), 173.66 (CH_3_C=O), 176.89 (COOH). *m/z* (ESI-MS+): 165.0 [M+H]^+^.

*Hyperpolarized ^13^C MRI*

35 μl of 3.2 M [1-^13^C] NAC with 17 mM OX063 was hyperpolarized using the SPINlab (GE Healthcare) for 3-4 hours, and the scans were performed using the Philips Achieva 3T MRI. ^13^C two dimensional spectroscopic chemical shift images (CSIs) were acquired with a 28x 28 mm, field of view in a 10 mm axial slice through the head, a matrix size of 14 x 14, spectral width of 3333 Hz, repetition time of 86 ms, and excitation pulse width a flip angle of 3^o^ for the mouse head, and with a 32 x 32 mm, field of view in a 10 mm coronal slice through the body, a matrix size of 16 x 16, spectral width of 3333Hz, repetition time of 85 ms, and excitation pulse with a flip angle of 10^o^ for the mouse body. CSIs were acquired 30 seconds after the beginning of the hyperpolarized [1-^13^C] NAC injections.

*LC/MS methods for identification of product*

Chemicals: [^13^C_3_, ^15^N]-NAC was purchased from Cambridge Isotope Laboratories, Inc (Tewksbury, MA). NAC, formic acid and ammonium formate was purchased from Sigma-Aldrich (St. Louis, MO). LC-MS acetonitrile was purchased from Fisher Scientific.

Liquid chromatography/mass spectrometry analysis was performed on a Waters Acquity UPLC coupled to a Waters Xevo Q-ToF quadruple time of flight mass spectrometer operating in electrospray ionization (ESI) in negative mode. The capillary and sampling cone voltages were set to 1.5 kV and 10 V, respectively. Source and desolvation temperatures were set to 120 ℃ and 450 ℃, respectively, and the cone and desolvation gas flows were set to 50.0 and 800.0 L/hour, respectively. To maintain mass accuracy, leucine enkephalin was used at a concentration of 2ng/mL in 50:50 acetonitrile/water containing 0.1% formic acid and injected at a rate of 10 μL/min. Data was acquired using SONAR (scanning quadrupole data-independent acquisition) in continuum mode. In low-energy MS1 mode, the quadrupole was scanned between 50 -1200 m/z, with a quadrupole transmission width of ~50 Da, with a collision cell energy of 10 eV. In high-energy MS2 mode, the collision cell energy was ramped between 20 – 30 Da. The analytes were separated by HILIC chromatography on an Xbridge BEH Amide (2.5 μm, 2.1 X 100 mm) column. Chromatographic separation was achieved with 95:5 water:acetontrile containing 10mM ammonium formate, pH 3 (A) and 95:5 acetonitrile:water containing 10 mM ammonium formate, pH 3 (B). Gradient elution, with a flow rate of 0.340 mL/min, began at 95% B, then decreased to 50% B from 0.0 to 3.4 minutes, 50-5% B from 3.4 to 5.39 minutes, held at 5% B from 5.39 to 6.37 minutes, then returned to initial conditions (95%B) in 0.20 minutes. The column was equilibrated at 95% B for 4.43 minutes before the next injection. The column temperature was maintained at 40°C in a column oven.

*Cell culture and animal studies*

All of the animal experiments were conducted in compliance with the Guide for the Care and Use of Laboratory Animal Resources, and experimental protocols were approved by the Animal Care and Use Committee, National Cancer Institute (NCI-CCR-ACUC).^1^ The human pancreatic ductal adenocarcinoma (PDAC) cell lines, Hs776t, and SU.86.86 cells, were purchased from Threshold Pharmaceuticals (Redwood City, CA). Human pancreatic tumor inoculated mice were generated by subcutaneous injection of 3×10^5^ cells into the right hind legs of mice. Detailed conditions for cell culture and xenograft tumor development were as described previously.^2^ Athymic nude mice were obtained from the Frederick Cancer Research Center, Animal Production (Frederick, MD). Both respiration (60–90 breaths per min) and temperature (35–37 °C) were maintained at a normal physiological range and monitored continuously during the animal experiment using the adjusted anesthesia. For all imaging studies, animals were anesthetized with isoflurane using a nose cone.

*Extraction of Metabolites from Tumors*

^13^C, ^15^N labeled NAC ([^13^C_3_, ^15^N] cysteine) was purchased from Cambridge Isotope Laboratories, Inc (Tewksbury, MA). Unlabeled NAC was purchased from Sigma-Aldrich (St. Louis, MO). 2.76 mg of either ^13^C, ^15^N labeled NAC ([^13^C_3_, ^15^N] cysteine) or unlabeled NAC was intravenously injected to track metabolites of NAC in xenograft tumors. Mice were euthanized in 2 minutes after the tail vein injections. The tumors were rapidly removed and flush frozen in the liquid nitrogen, then they were stored at -80^o^C. The metabolites were extracted from the obtained tumors using a previously reported procedure.^3^ The resulting lyophilized aqueous metabolite extracts were used for the MS for metabolomic analysis.

*Preparation/synthesis of model compounds*

Cysteinylglycine, Cys-Gly, was purchased from Sigma-Aldrich (St. Louis, MO).

**[1-^13^C] *N*-Acetyl cysteine-[1-^13^C] *N*-acetyl cysteine 3 and [1-^13^C] *N*-acetyl cysteine-Glutathione 4:** [1-^13^C] NAC **1** (0.0257 g, 0.156 mmole) and GSH (0.0962 g, 0.313 mmole) powders were combined in a vial. To this mixture, a 10 % solution of DMSO in DI water (1.0 mL) was added. The resulting mixture was gently shaken for 5 minutes until a clear solution was obtained and the vial was exposed to air for 16 h at RT. LC-MS analysis indicated presence of GSH-[1-^13^C] NAC **3**, [1-^13^C] NAC-[1-^13^C] NAC **4** and GSH-GSH **5** dimers. Products **3** and **4** were observed to be in roughly equal proportion while **5** was the major product. Reaction mixture was diluted with 20 mL DI water and lyophilized to obtain solid powder. Separation of various products was performed using an Agilent Prep C18 column (5 µm, 50 x 100 mm) with a flow rate of 50 mL/min. A linear gradient of 0-25 % acetonitrile with 0.05 % TFA was used to elute the products, all obtained as white powder after lyophilization.

**[1-^13^C_2_] L-Cystine 6:** [1-^13^C] L-cysteine **2** (0.010 g, 0.08 mmol) was dissolved in 10 % solution of DMSO in DI water (250 μl) and gently shaken for 5 minutes until a clear solution was obtained. It was then exposed to air for 16 h at RT. Analysis by LC-MS indicated a complete conversion of the starting material to L-Cystine **6**. Reaction mixture was diluted with 5 ml of DI water and lyophilized to obtain **6** a white powder.

**Supplemental Figure S1**


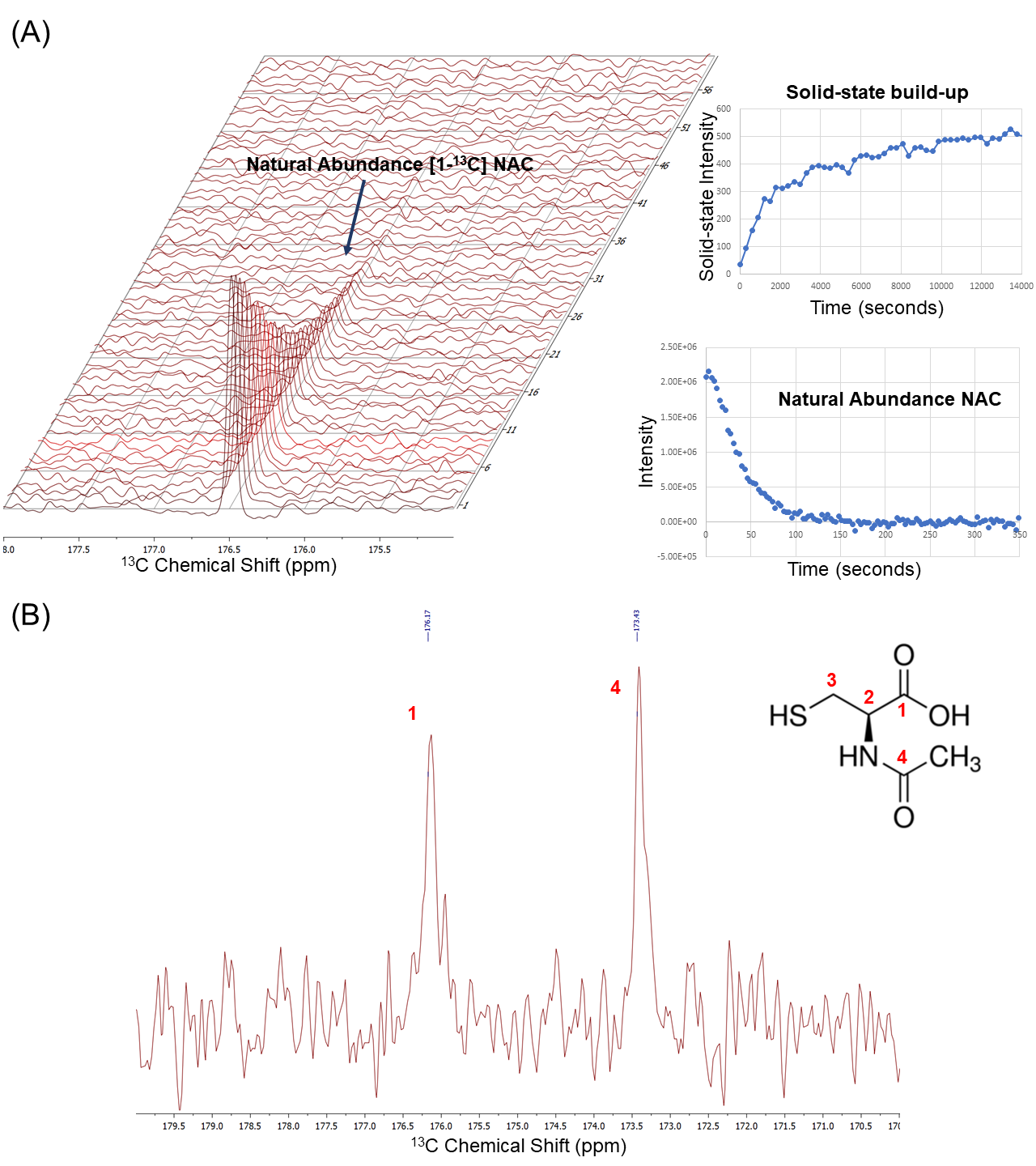


**Figure S1.** (A) Dynamic ^13^C MR spectra of hyperpolarized natural abundance NAC on 1T NMR spectrometer indicated that an only [1-^13^C] NAC signal can be observed in the hyperpolarized ^13^C NMR spectra, although NAC has two carbonyl groups in the chemical structure, including **1**: [1-^13^C] and **4**: [4-^13^C], as shown in the thermal NMR of natural abundance NAC at 1T NMR spectrometer (B).

**
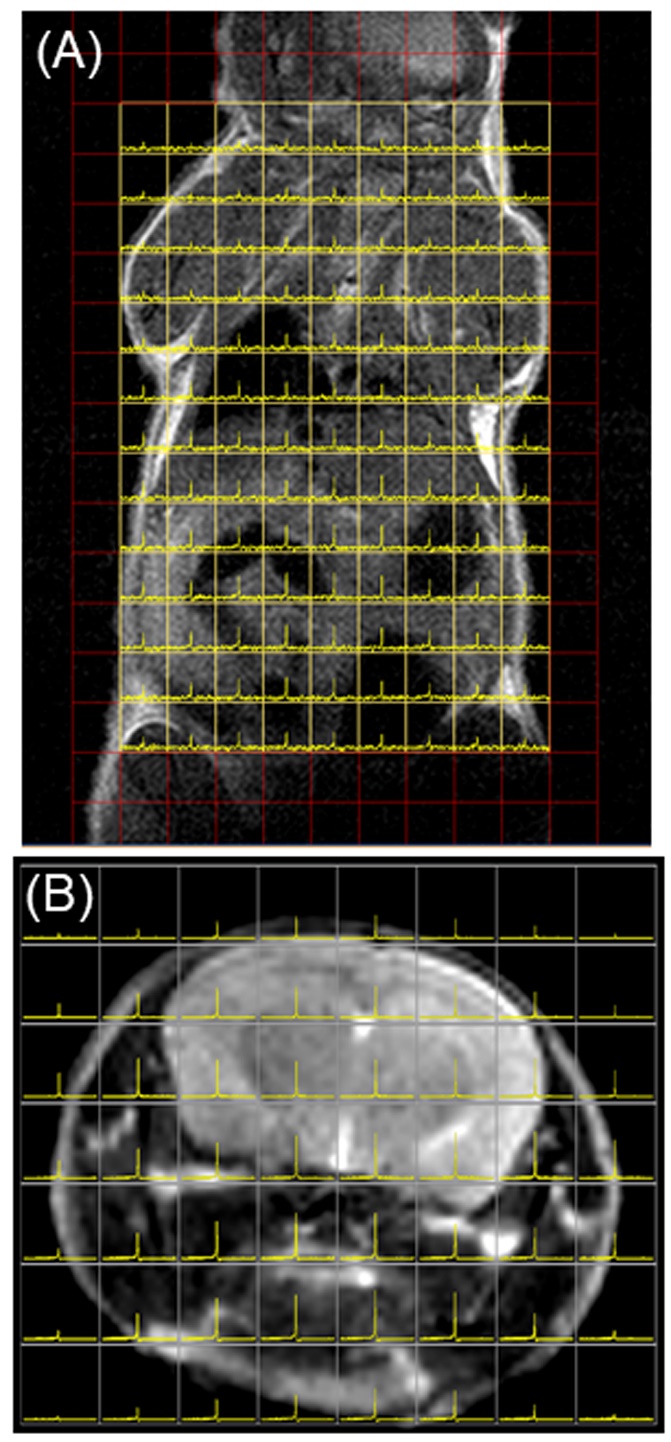
Supplemental Figure S2**

**Figure S2.** (A) Broad distribution of hyperpolarized [1-^13^C] NAC in the mouse body observed by ^13^C Chemical Shift Imaging (CSI). (B) Hyperpolarized ^13^C CSI in the mouse head acquired within 30 seconds after the injection of hyperpolarized [1-^13^C] NAC. The CSI was obtained from 10 mm thickness slice and overlaid on a 2 mm thickness of *T_2_*-weighted anatomical images. ^13^C CSI in mouse head acquired using Philips 3T scanner after hyperpolarized ^13^C NAC injection. *T_2_*-weighted anatomical images were acquired using turbo spin echo with the following parameters: FOV 28 mm, TR 2500 ms, resolution 0.19 x 0.19 mm, slice thickness 2 mm, 14 slices. 35 µL of 880 mg/mL ^13^C NAC containing 17 mM OX063 in 5M NaOH was polarized using SPINlab polarizer for 4 hours and was dissolved in 4.5 mL PBS containing 100 mg/mL EDTA. 300 µL of hyperpolarized ^13^C NAC was injected intravenously to a nude mouse, and ^13^C CSI was acquired 30-44 sec after the injection with FOV 28 mm, TR 86 ms, FA 10 deg, spectral width 3330 Hz, matrix size 14 x 14, and slice thickness 10 mm.

**Supplemental Figure S3**


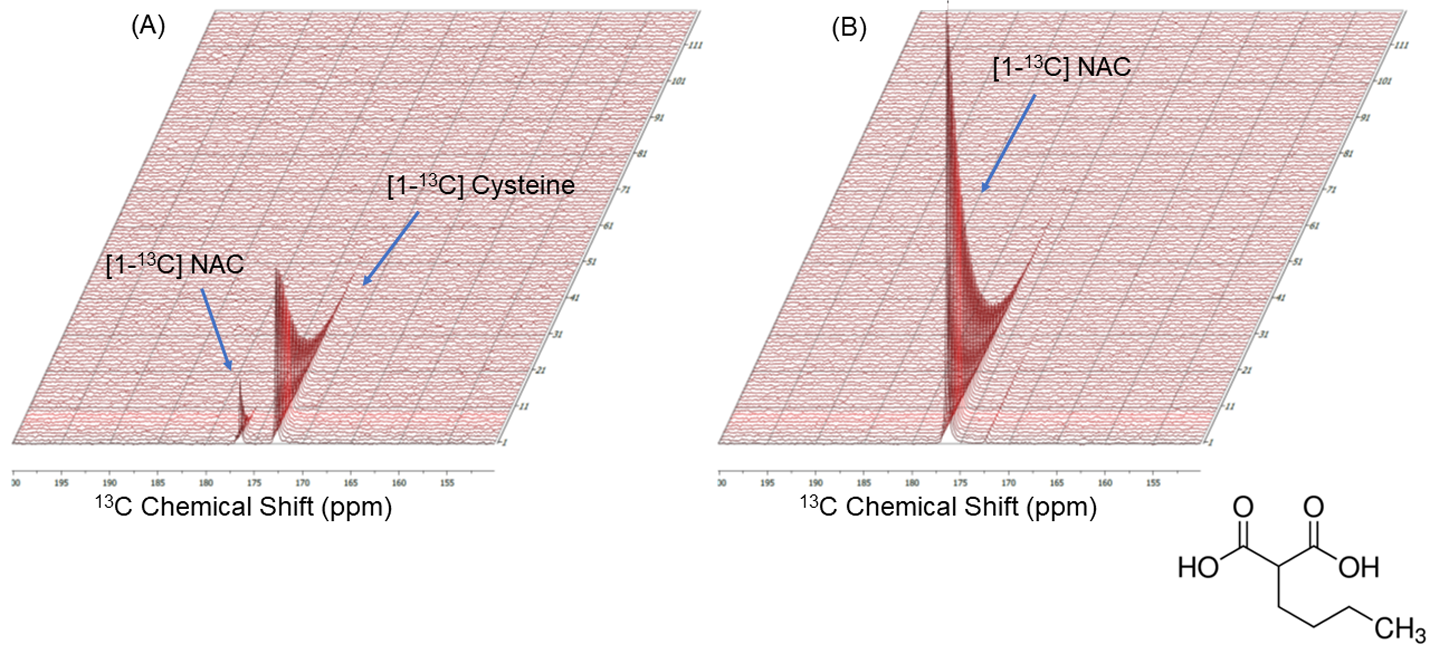


**Figure S3.** Hyperpolarized ^13^C NAC enzymatic assays with acylase 1 without (A) and with (B) an inhibitor, n-Butylmalonic acid on 1T NMR spectrometer.

**Supplemental Figure S4**


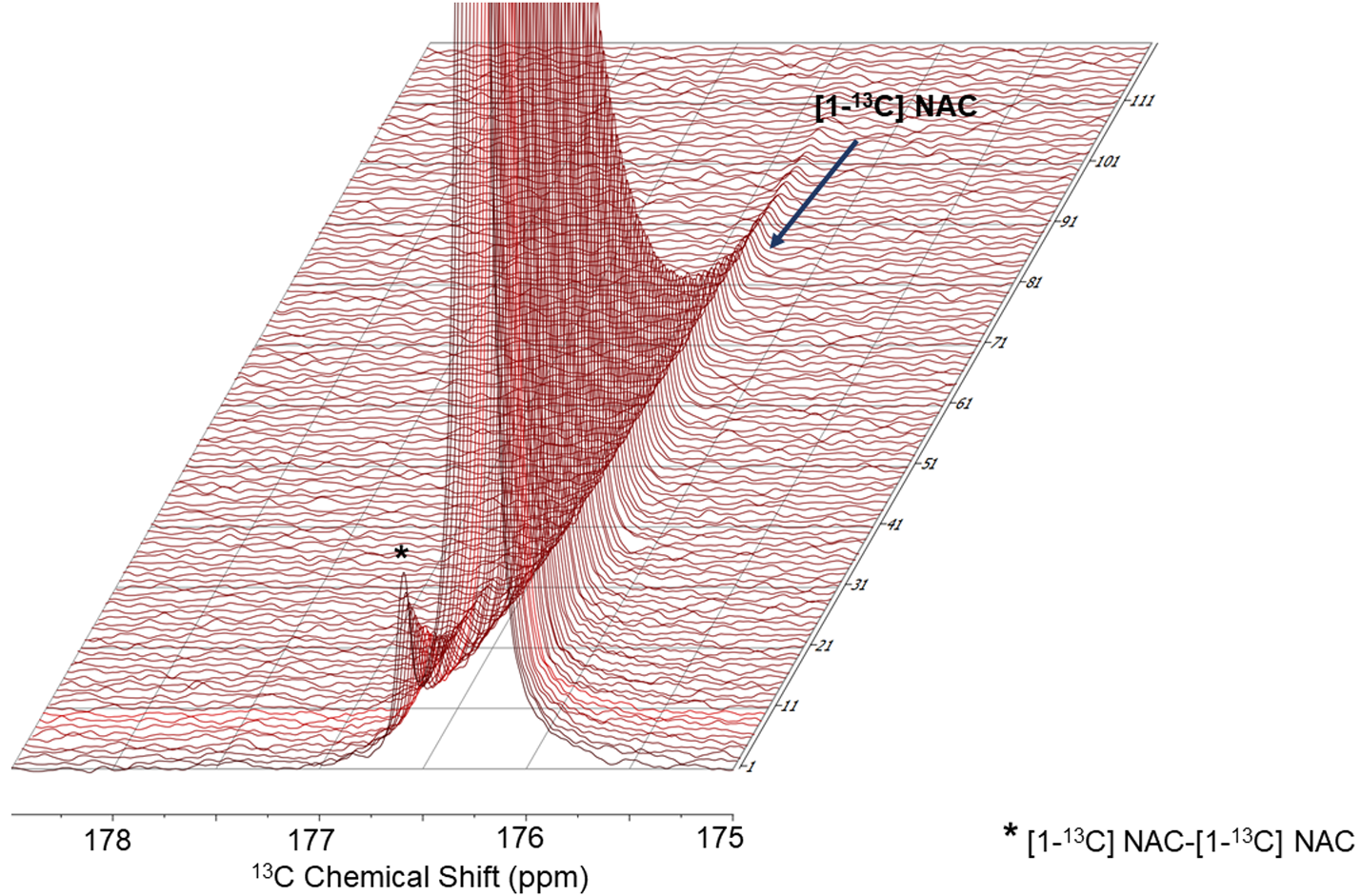


**Figure S4.** The dynamic ^13^C MRS of hyperpolarized [1-^13^C] NAC on 1T NMR spectrometer. In addition to major [1-^13^C] NAC peak, the minor signal of [1-^13^C] NAC-[1-^13^C] NAC was observed with optimized shimming conditions.

**Supplemental Figure S5**


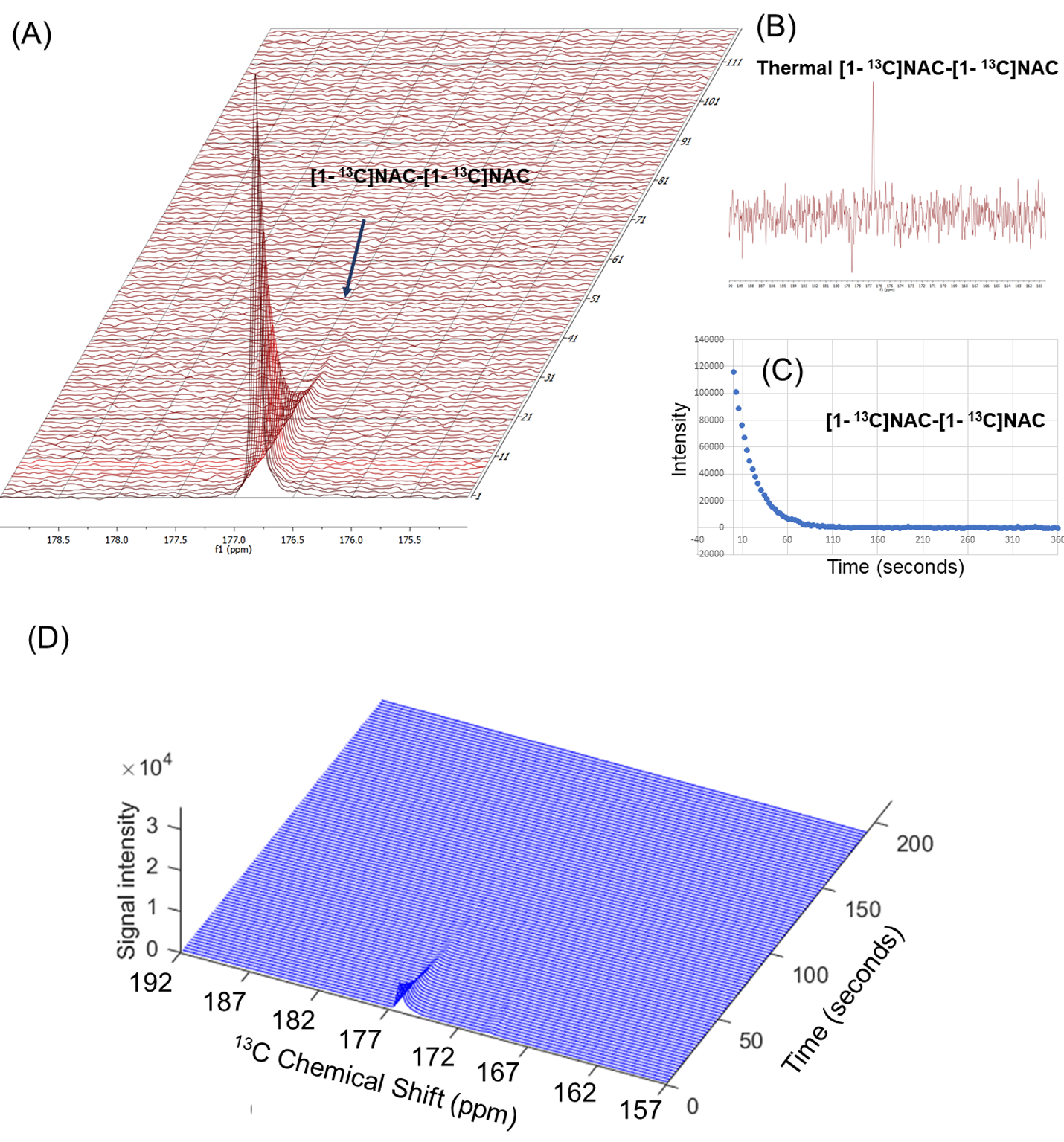


**Figure S5.** The dynamic ^13^C MRS of hyperpolarized [1-^13^C] NAC-[1-^13^C] NAC at pH = 7 on 1T NMR spectrometer (A). Thermal spectra of [1-^13^C] NAC-[1-^13^C] NAC was observed on 1T NMR with 16384 scans, 5 mM ProHance (B). The decay of dynamic ^13^C MR signal of hyperpolarized [1-^13^C] NAC-[1-^13^C] NAC on 1T NMR spectrometer (C). The dynamic ^13^C MRS of hyperpolarized [1-^13^C] NAC-[1-^13^C] NAC at pH = 7 on 3T NMR spectrometer (D).

**Supplemental Figure S6**


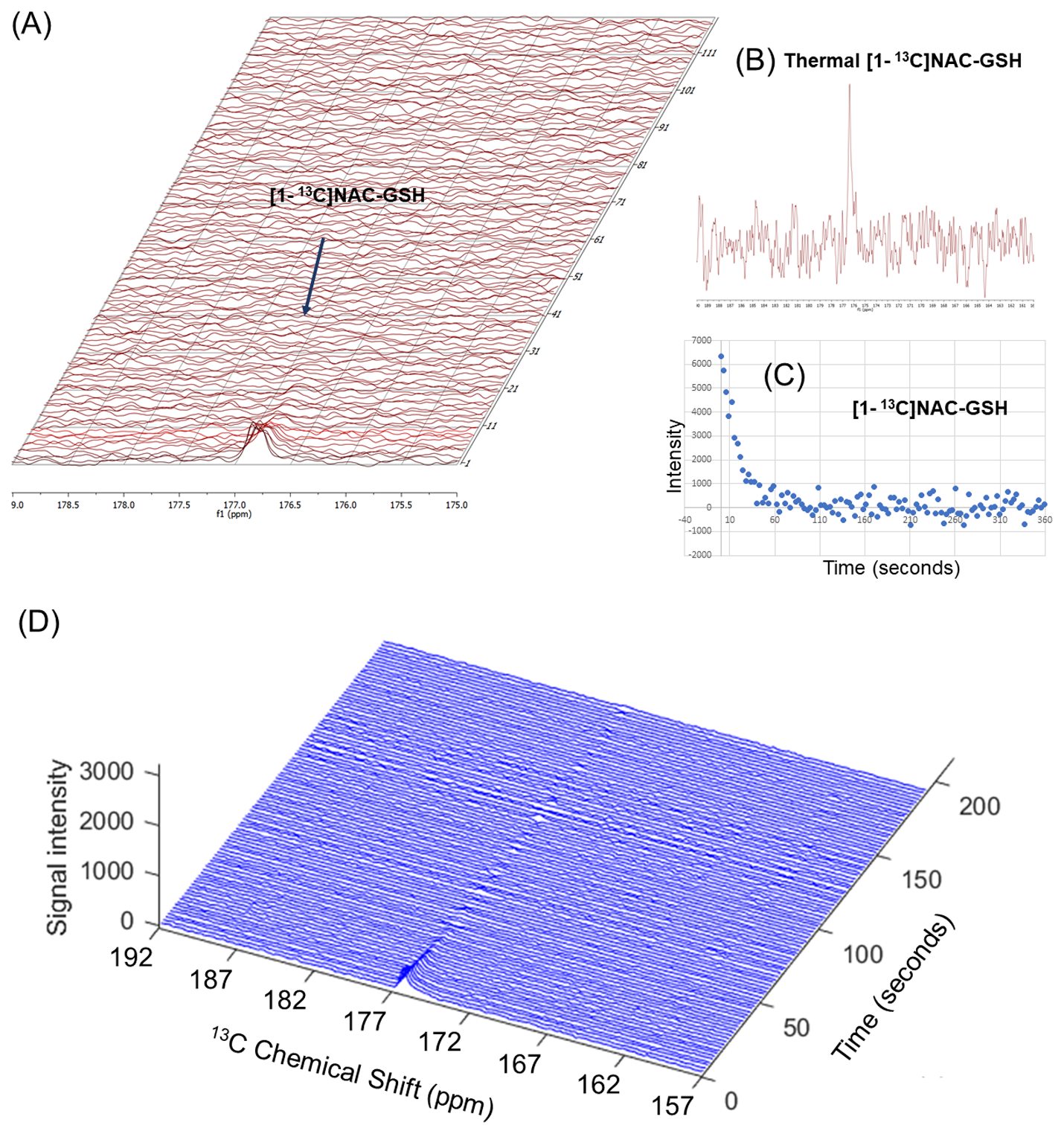


**Figure S6** The dynamic ^13^C MRS of hyperpolarized [1-^13^C] NAC-GSH at pH = 7 on 1T NMR spectrometer (A). Thermal spectra of [1-^13^C] NAC- GSH was observed on 1T NMR with 43000 scans, 5 mM ProHance (B). The decay of dynamic ^13^C MR signal of hyperpolarized [1-^13^C] NAC- GSH on 1T NMR spectrometer (C). The dynamic ^13^C MRS of hyperpolarized [1-^13^C] NAC- GSH at pH = 7 on 3T NMR spectrometer (D).

**Supplemental Figure S7**


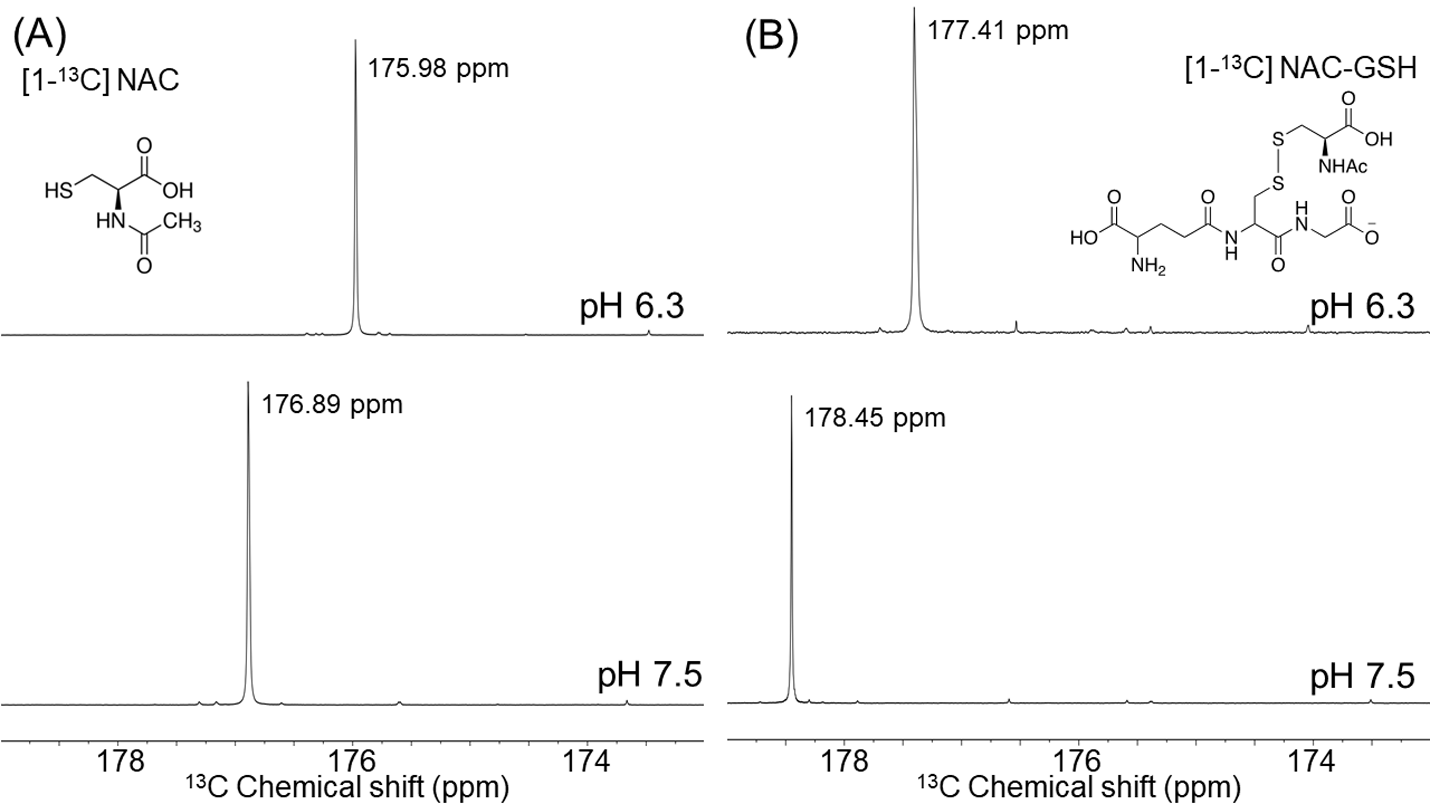


**Figure S7.** Both NAC (A) and its reaction products, NAC-GSH (B), have pH dependence of ^13^C chemical shifts on well optimized shimming conditions. The chemical shift differences were demonstrated at pH 7.5 (bottom) and pH 6.3 (top).

**Supplemental Figure S8**


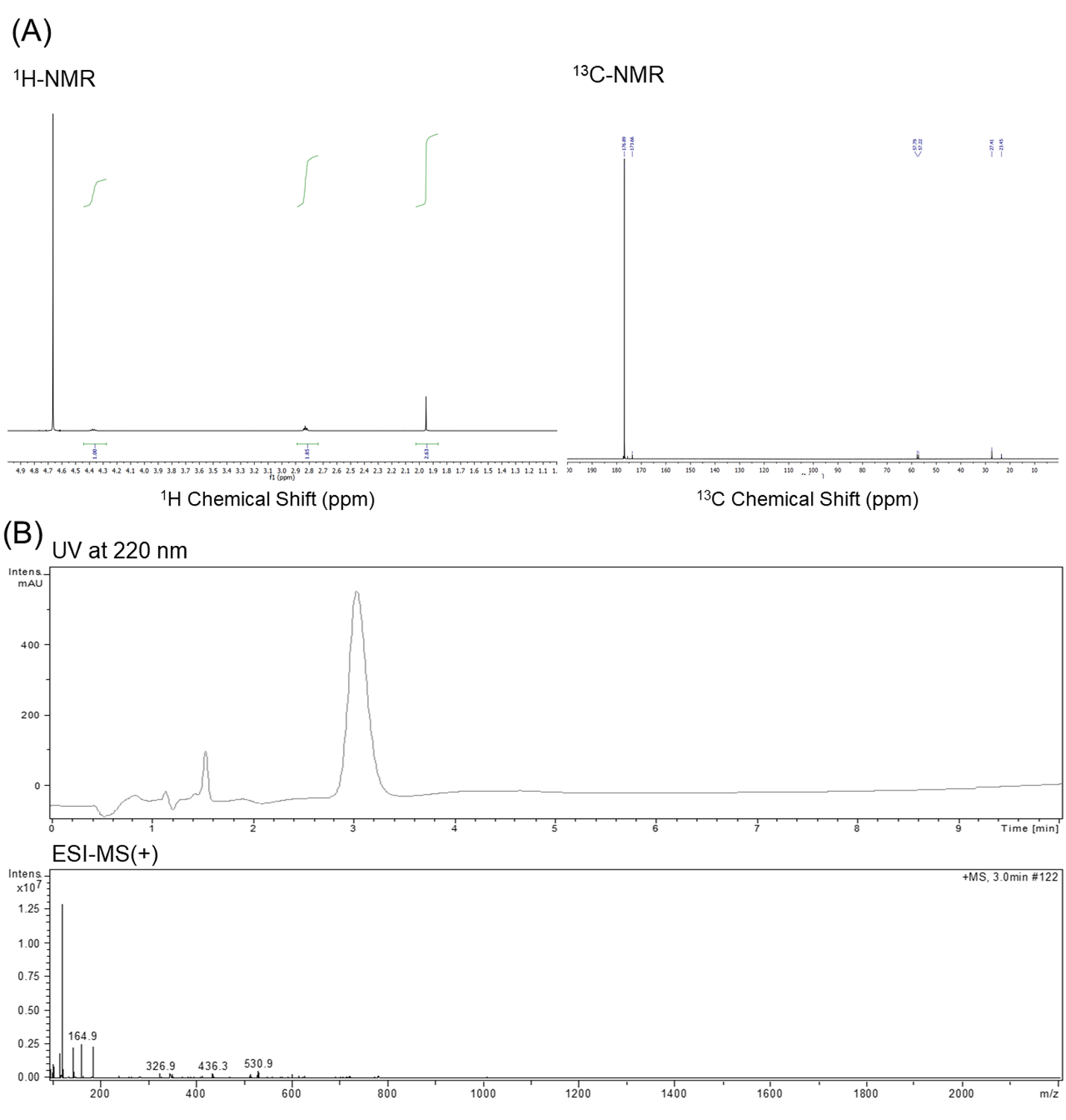


**Figure S8.** Evaluation of synthesized [1-^13^C] NAC. (A) ^1^H NMR (left) and ^13^C NMR (right), (B) Separation and analysis on RP-HPLC (top) and ESI-MS (bottom).


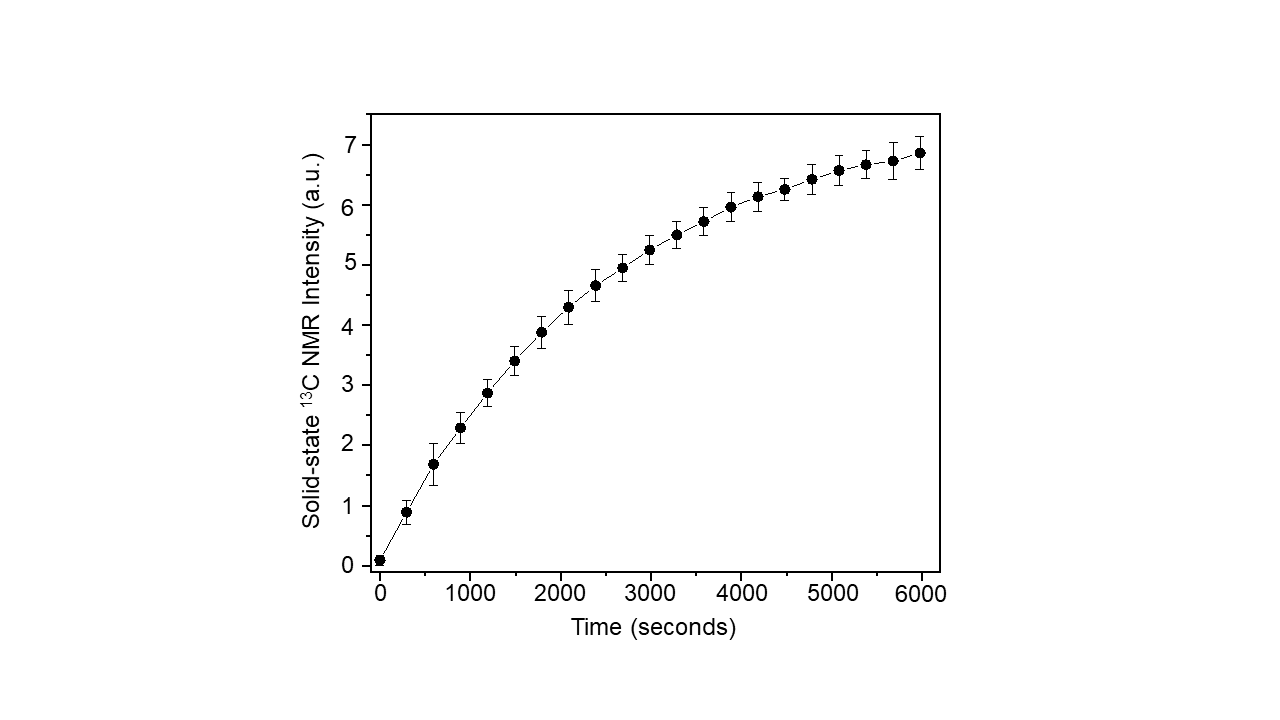


**Figure S9.** Hyperpolarization build-up curves of [1-^13^C] NAC (*n* = 3; error bars, SD) showing the consistent polarization on the Hypersense hyperpolarizer using the optimized condition of a NaOH solution.

**References**

1. National Research Council, *Committee for the Update of the Guide for the Care and Use of Laboratory Animals. Guide for the care and use of laboratory animals*. (Washington, D.C.: Institute of Laboratory Animal Resources, 2011).
2. Yamamoto, K. *et al*. Molecular Imaging of the Tumor Microenvironment Reveals the Relationship between Tumor Oxygenation, Glucose Uptake, and Glycolysis in Pancreatic Ductal Adenocarcinoma. *Cancer Res*. **80**, 2087-2093 (2020).
3. Crooks, D. R., Fan, T. W. & Linehan, W. M. Metabolic Labeling of Cultured Mammalian Cells for Stable Isotope-Resolved Metabolomics: Practical Aspects of Tissue Culture and Sample Extraction. *Methods. Mol. Biol.* **1928**, 1-27 (2019).
